# Follow-up after pyeloplasty: How long?

**Published:** 2008

**Authors:** T. J. Nirmal, J. C. Singh

**Affiliations:** Department of Urology, Christian Medical College, Vellore, India. E-mail: nirmaltj@yahoo.com

## SUMMARY

This was a retrospective study of 138 patients who underwent a successful dismembered pyeloplasty over an eight-year period.[1] Patients were divided into three groups based on the duration of follow-up with renal scans. Group one (138) had a renal scan at a mean of nine months after surgery and the split renal function (SRF) before and after surgery was compared. Group two (35) had a second scan at 3.5 years after surgery and group three (29), in addition, had another scan at 5.5 years. The SRF of the scan after surgery and the late scan at 3.5 and 5.5 years were compared. A change in SRF of greater than 5% was considered significant. The mean (range) SRF was marginally better in all three groups at follow-up. Repeat renal scans at 3.5 and 5.5 years after surgery showed stable SRF, even if the renal function was already diminished. Of 138 patients, only five had a significant deterioration in SRF to less than 40%. Hence, the authors have concluded that repeat renal scans in a five to seven-year period after pyeloplasty don't seem to be justified, as most renal units remain stable.

## COMMENTS

Dismembered Anderson-Hynes pyeloplasty is a successful treatment for ureteropelvic junction (UPJ) obstruction with success rates as high as 98%.[2] Long-term data in adults has shown five to 15-year durability of success.[3] For these reasons, dismembered pyeloplasty remains the first line surgical procedure for the majority of pediatric urologists.

Defining a true UPJ obstruction in the pediatric population remains difficult. Serial ultrasounds, measurement of resistive index (RI) using duplex ultrasonography and intravenous urography are few of the various modalities used. Radionuclide renography is one of the modalities with objective measurements. Calculation of SRF and assessing wash out curves is important in the diagnosis and follow-up. However, there are only a few reports in children on how long these patients need follow-up. Also, little is known about the long-term renal function on consecutive renal scans, especially whether loss of renal function in the absence of obstruction might progress with time. The authors have attempted to answer some of these questions.

Apart from the retrospective design, one of the major drawbacks of this study is that the majority of the patients with an immediate postoperative SRF more than 40% were discharged from follow-up assuming that their renal function would remain stable. Hence, only 29 of the 138 patients had follow-up scans at a mean of 5.5 years.

O'Reilly *et al*. performed a repeat renal scan in 24 patients at 6-19 years after surgery and concluded that the results were durable.[4] Another study by Boubaker *et al*. showed that after an unobstructed diuretic renogram, recurrence of the obstruction was unlikely and did not justify a long-term follow-up.[5]

One of the concerns advocating follow-up of post pyeloplasty children is the early detection of renal functional loss. The use of SRF as a tool for follow-up may not actually reflect the natural course of previously obstructed kidneys as a decrease in SRF might be either due to deterioration of the operated kidney or due to contralateral compensation. This study had no data to estimate absolute single-kidney function and therefore no firm conclusions could be drawn about the recovery or deterioration of the affected kidneys.

Boubaker *et al*. using accumulation index (AI) as an indicator of absolute function, reported that in 53 children who had surgery because of presumed UPJ obstruction, the AI improved in 88% after 5-15 years of follow-up; the improvement was most notable in those with impaired renal function before surgery.[6]

In conclusion, this study does show that most renal units remain stable after a successful pyeloplasty. But further studies are required to investigate whether previously obstructed kidneys will deteriorate in time, and to determine which patients with poorly functioning kidneys after pyeloplasty are at risk of developing hypertension, infection, proteinuria or pregnancy-related problems.
